# The Río Hortega University Hospital Glioblastoma dataset: A comprehensive collection of preoperative, early postoperative and recurrence MRI scans (RHUH-GBM)

**DOI:** 10.1016/j.dib.2023.109617

**Published:** 2023-09-23

**Authors:** Santiago Cepeda, Sergio García-García, Ignacio Arrese, Francisco Herrero, Trinidad Escudero, Tomás Zamora, Rosario Sarabia

**Affiliations:** a Department of Neurosurgery, Río Hortega University Hospital, Dulzaina 2, 47012 Valladolid, Spain; b Department of Radiology, Río Hortega University Hospital, Dulzaina 2, 47012 Valladolid, Spain; c Department of Pathology, Río Hortega University Hospital, Dulzaina 2, 47012 Valladolid, Spain

**Keywords:** MR imaging, Neuro-oncology, Radiology, Brain tumor

## Abstract

Glioblastoma, a highly aggressive primary brain tumor, is associated with poor patient outcomes. Although magnetic resonance imaging (MRI) plays a critical role in diagnosing, characterizing, and forecasting glioblastoma progression, public MRI repositories present significant drawbacks, including insufficient postoperative and follow-up studies as well as expert tumor segmentations. To address these issues, we present the “Río Hortega University Hospital Glioblastoma Dataset (RHUH-GBM),” a collection of multiparametric MRI images, volumetric assessments, molecular data, and survival details for glioblastoma patients who underwent total or near-total enhancing tumor resection. The dataset features expert-corrected segmentations of tumor subregions, offering valuable ground truth data for developing algorithms for postoperative and follow-up MRI scans.

Specifications Table

**Table:** 

Subject	Health and Medical Sciences: - Medical Imaging - Oncology - Surgery
Specific subject area	Magnetic resonance imaging (MRI) data and tumor segmentations from patients with glioblastoma.
Data format	Raw
Type of data	The dataset contains MRI data including the sequences: T1-weighted (T1w), T2-weighted (T2w), fluid attenuated inversion recovery (FLAIR), T1w contrast-enhanced (T1ce), and diffusion-weighted imaging-derived apparent diffusion coefficient (ADC) maps. Data is available in both raw DICOM and processed NIfTI format along with expertly refined tumor segmentations. Clinical information is also available in CSV format.
Data collection	MRI data were collected retrospectively from the hospital's picture archiving and communication system (PACS). Imaging data were acquired on a 1.5 Tesla MRI scanner and consists of multiparametric structural and diffusion MRI images acquired at three time points: preoperatively, early, and at follow-up when tumor recurrence was diagnosed. In addition, the dataset includes only patients who underwent total or near-total resection of the enhancing tumor. Clinical data were collected from electronic medical records. Tumor subregion segmentations were generated by computer aided methods and carefully reviewed and manually corrected by two expert neurosurgeons specializing in neuroimaging.
Data source location	The Río Hortega University Hospital, Valladolid, Spain.
Data accessibility	Repository name: The Cancer Imaging Archive Data identification number: https://doi.org/10.7937/4545-c905 Direct URL to data: https://wiki.cancerimagingarchive.net/pages/viewpage.action?pageId=145755234 Instructions for accessing these data: Raw DICOM data in this collection contains images that could potentially be used to reconstruct a human face. To safeguard the privacy of participants, users must sign and submit a TCIA Restricted License Agreement

## 1 Value of the Data

• The value of The Río Hortega University Hospital Glioblastoma Dataset (RHUH-GBM) [1] lies in the inclusion of longitudinal MRI scans obtained at critical points in the disease course: pre-surgery, early post-surgery, and at the time of recurrence. It is important to note that patients in this cohort underwent either gross total or near-total tumor resection, further enhancing the dataset's significance. Moreover, the dataset features meticulously refined expert segmentations, contributing to its overall richness.
• Researchers and medical professionals alike can leverage this dataset for a wide range of research purposes. These applications include enhancing automatic segmentation algorithms tailored to brain tumor postoperative scans, developing models for predicting survival rates, and investigating recurrence patterns in patients who have undergone complete tumor resection.
• Furthermore, the dataset is accessible in two distinct formats: NiFTI and DICOM, and it is readily available on the TCIA website. Additionally, the accompanying clinical data is comprehensive, encompassing demographic, pathological, radiological, volumetric, and survival information, further enriching its utility for research endeavors.

## 2 Data Description

### 2.1 Shared files

Table 1 shows the details of the files available through TCIA. DICOM and NiFTI files can be visualized in dedicated and publicly available software such as 3D Silicer (www.slicer.org) and ITK-SNAP (www.itksnap.org). Clinical data is also available in CSV format.

**Table 1:** Detailed dataset description.

Number of Patients	40
Number of Studies	120
Number of Series	600
Number of Raw Images	37,425 (DICOM)
Number of Processed Images + Tumor segmentations	720 (NIfTI)
Images Size (GB)	16 GB (DICOM) + 2.9 GB (NIfTI)

### 2.2 Patient population

The dsataset comprises consecutive patients who underwent surgery between January 2018 and December 2022, with a confirmed histopathological diagnosis of WHO grade 4 astrocytoma. Forty patients were selected based on the following inclusion criteria: 1) Gross total resections (GTR) or Near Total Resection (NTR), defined as having no residual tumor enhancement and an extent of resection exceeding 95% of the initial enhancing volume, respectively [2,3]. 2) Availability of MRI studies at three time points: preoperative, early postoperative (within 72 h), and the follow-up scan where tumor progression was diagnosed. 3) Availability of structural T1-weighted (T1w), T2-weighted (T2w), T1 contrast-enhanced (T1ce), Fluid-attenuated inversion recovery (FLAIR), and diffusion-weighted imaging-derived apparent diffusion coefficient (ADC) maps for each study. 4) Receipt of adjuvant treatment with chemotherapy and radiotherapy following the Stupp protocol [4]. Patients with severe image acquisition artifacts or missing MRI series were excluded. The modified Response Assessment in Neuro-Oncology (RANO) criteria were utilized to determine tumor progression [5].

A summary of the demographic data is presented in Table 2. The patients had an average age of 63 ± 9 years, consisting of 28 men (70%) and 12 women (30%). The median preoperative Karnofsky Performance Scale (KPS) score was 80. Out of the 40 patients, 38 (95%) were diagnosed with de novo glioblastomas, while two patients (5%) had recurrent glioblastomas previously treated with standard chemoradiotherapy. Four cases (10%) were IDH-mutated, and 36 cases (90%) were IDH wild-type.

**Table 2:** Study population demographics of the Río Hortega University Hospital Glioblastoma dataset (RHUH-GBM).

Sex	Male	28 (70%)
	Female	12 (30%)
Age (years)	63 ± 9	
Extent of resection	GTR	27 (67.5%)
	NTR	13 (32.5%)
Number of time-point MRI studies	120	
Number of MRI series	600	
IDH status	Mutant	4 (10%)
	Wild type	36 (90%)
Preoperative KPS	80 (10)	
Operative adjuncts	5’ALA	40 (100%)
	Sodium Fluorescein	7 (17.5%)
	Neuronavigation	40 (100%)
	ioUS	40 (100%)
	IONM	4 (10%)
	DES	3 (7.5%)
Preoperative contrast enhancing tumor volume (cm^3^)	34.99 ± 26.59	
Preoperative T2/FLAIR peritumoral signal alteration volume (cm^3^)	35.00 ± 26.74	
Postoperative contrast enhancing tumor volume (cm^3^)	0.23 ± 0.47	
Postoperative T2/FLAIR peritumoral signal alteration volume (cm^3^)	35.00 ± 26.74	
Radiotherapy treatment details	VMAT-IMRT-IGRT/60 Gy /30 fx	29 (72.5%)
	VMAT-IMRT-IGRT/50 Gy /20 fx	6 (15%)
	VMAT-IMRT-IGRT/40.5 Gy /15 fx	5 (12.5%)
Postoperative neurological deficit	No	26 (65%)
	Transient	6 (15%)
	Minor persistent	6 (15%)
	Major persistent	2 (5%)
Postoperative KPS	80 (20)	

Numerical values are expressed in mean and standard deviation or median and interquartile range accordingly. GTR = gross total resection, NTR = near total resection, IDH = isocitrate dehydrogenase, 5’ALA = 5-aminolevulinic acid, ioUS = intraoperative ultrasound, IONM = Intraoperative Neurophysiological Monitoring, DES = direct electrical stimulation, VMAT = Volumetric Modulated Arc Therapy, IMRT = Intensity Modulated Radiation Therapy, IGRT = Image-Guided Radiation Therapy. Radiotherapy treatments are expressed in dose (Gy) and number of fractions (fx).

**Fig. 1:**
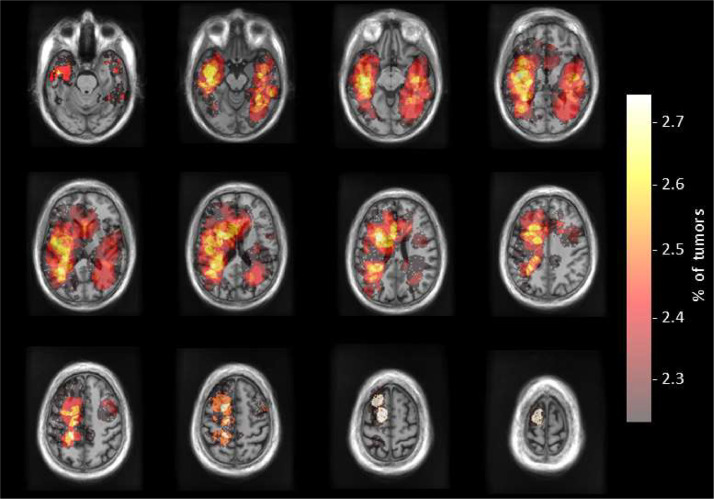
A graphical representation of tumor location is presented in the form of a heatmap, showcasing the distribution of tumors in a normalized SRI24 atlas template space. Areas of interest are depicted as percentages.

The mean preoperative contrast-enhancing tumor volume was 34.99 ± 26.59 cm^3^, and the mean postoperative contrast-enhancing residual tumor volume was 0.23 ± 0.47 cm^3^. A graphical representation of tumor location is displayed as a heatmap in Fig. 1. Among the patients, 27 (67.5%) underwent gross total resection, and 13 (32.5%) underwent near-total resection. The median overall survival was 364 days, and the median progression-free survival was 198 days.

### 2.3 Clinical, pathological, and imaging data

Clinical and pathological information was obtained from electronic medical records, including age, sex, histopathological diagnosis, pre- and postoperative Karnofsky Performance Score (KPS), isocitrate dehydrogenase (IDH) status, use of operative adjuncts, volumetric assessment of the extent of resection of the contrast-enhancing and non-enhancing tumor, presence of postoperative neurological deficits, details of chemotherapy and radiotherapy received, and overall survival (OS) and progression-free survival (PFS) times. OS was measured from diagnosis to death or last follow-up if alive, while PFS was from diagnosis to tumor progression or last follow-up if no progression was noted. Out of the total sample, a subset of 11 patients had initially undergone preoperative and subsequent follow-up MRI scans at a secondary healthcare facility before being referred to the primary center. Details of the MR imaging acquisition parameters are described in Table 3.

**Table 3:** MRI acquisition parameters.

		Primary center	Secondary center	
Manufacturer, model, and Field strength		General electric, signa HDxT, 1.5 T	Philips, ingenia ambition X, 1.5 T	Philips, achieva, 1.5 T
Number of MRI studies		107 (89 %)	11 (9 %)	2 (2 %)
MRI sequence	T1ce	TR/TE, 7.98 ms/2.57 ms; 3D; GRE; matrix, 512 × 512; slice thickness, 1 mm	TR/TE, 17.96 ms/6.43 ms; 3D ProSET; matrix, 230 × 230; slice thickness, 1 mm	TR/TE, 25 ms/6.7 ms; ProSET, 3D; matrix, 256 × 256; slice thickness, 1.6 mm
	T1w	TR/TE, 580 ms/7.56 ms; 2D; FSE; matrix, 512 × 512; slice thickness, 5 mm	TR/TE, 525.6 ms/12 ms; 2D; SE; matrix, 228 × 227; slice thickness, 5 mm	TR/TE, 456.2 ms/12 ms; 2D; SE; matrix, 249 × 191; slice thickness, 6 mm
	T2w	TR/TE, 5220 ms/96.12 ms; 2D; FRSE; matrix, 512 × 512; slice thickness, 5 mm.	TR/TE, 5327.3 ms/110 ms; 2D; TSE; matrix, 232 × 232; slice thickness, 3 mm.	TR/TE, 2456.2 ms/110 ms; 2D; TSE; matrix, 264 × 203; slice thickness, 5 mm.
	FLAIR	TR/TE, 8002 ms/135.07 ms; 2D; FSE; matrix, 512 × 512; slice thickness, 4 mm	TR/TE, 5000 ms/375.8 ms; 3D; SPIR; matrix, 196 × 196; slice thickness, 1.2 mm	TR/TE, 6000 ms/120 ms; 2D; FSE; matrix, 200 × 159; slice thickness, 2.8 mm
	DWI	TR/TE, 8000 ms/111.7 ms; matrix, 128 × 160; slice thickness, 5 mm; b-values, 0 and 1000 s/mm2	TR/TE, 4600 ms/84.4 ms; matrix, 190 × 190; slice thickness, 5 mm; b-values, 0 and 1000 s/mm2	TR/TE, 3414 ms/88.8 ms; matrix, 112 × 89; slice thickness, 5 mm; b-values, 0 and 1000 s/mm2

T1ce = contrast-enhanced T1w, T2w= T2-weighted image, FLAIR = Fluid-attenuated inversion recovery, DWI = diffusion weighted image, TR = repetition time, TE= echo time, GRE = gradient echo. FSE= fast spin echo. FRFSE= fast recovery fast spin echo. ProSET= principle of selective excitation technique. SE= spin echo. SPIR= spectral presaturation with inversion recovery. TSE= turbo spin echo.

## 3 Experimental Design, Materials and Methods

### 3.1 Image preprocessing

Images were retrieved from the Picture Archiving Communication System (PACS) in Digital Imaging and Communications in Medicine (DICOM) format for subsequent processing. The first step involved converting the images to Neuroimaging Informatics Technology Initiative (NIfTI) format using the dicom2niix tool version v1.0.20220720 [6], available at https://github.com/rordenlab/dcm2niix/releases/tag/v1.0.20220720. Subsequently, the T1ce scans for each subject were registered to the SRI24 anatomical atlas space [7] using the FLIRT (FMRIB's Linear Image Registration Tool) [8,9] available at https://fsl.fmrib.ox.ac.uk/fsl/fslwiki/FSL. The T1w, T2w, FLAIR scans, and ADC maps were then registered to the transformed T1ce scan, resulting in co-registered resampled volumes of 1 × 1 × 1 mm isotropic voxels. The brain was extracted from all co-registered scans using a deep learning tool called Synthstrip [10], included in FreeSurfer v7.3.0, available at https://github.com/freesurfer/freesurfer/tree/dev/mri_synthstrip. Finally, intensity Z-scoring normalization was performed for structural MRI sequences (T1ce, T2w, T1w, and FLAIR) using the normalization tools included in Cancer Imaging Phenomics Toolkit (CaPTk) v1.9.0 [11] available at https://www.nitrc.org/projects/captk/. Intensity normalization was not performed on ADC maps since intensity values are already standardized and expressed in units of mm2/s. The code applied for image preprocessing is available to the public through a GitHub repository https://github.com/smcch/RHUH-GBM-dataset-MRI-preprocessing.

### 3.2 Tumor Subregions Segmentations

**Fig. 2:**
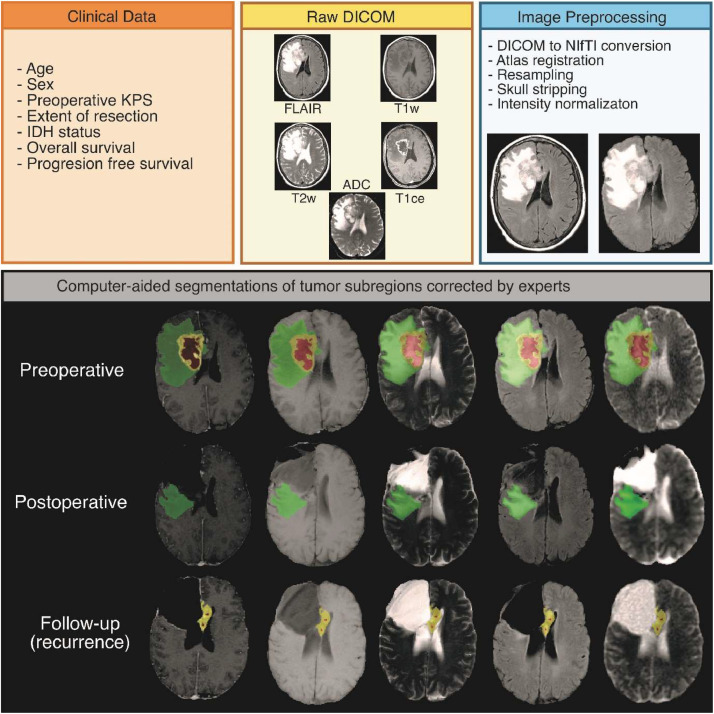
Schematic representation of the workflow. It displays the main clinical variables collected, the image preprocessing steps, and the results of the tumor subregion segmentations: red = necrosis, green = peritumoral region (T2/FLAIR) signal alteration, yellow = enhancing tumor.

The preprocessed images from each time point were used as input for generating computer-aided segmentations using Deep-Medic [12]. Three labels were subsequently obtained, corresponding to 1 - necrosis, 2 - peritumoral signal alteration, including edema and non-enhancing tumor, and 3 - enhancing tumor. All segmentations were carefully reviewed and manually corrected by two expert neurosurgeons specializing in neuroimaging (S.C. and S.G.). A summary of the data workflow is depicted in Fig. 2.

## Limitations

Not applicable.

## Ethics Statement

The study was conducted following the principles of the Declaration of Helsinki. Written consent was obtained from all patients, and approvals were granted by the Institutional Review Board of Río Hortega University Hospital and the Ethics Committee for Drug Research (CEIm) of the West Valladolid Health Area (Ref. 22PI-208).

## CRediT authorship contribution statement

**Santiago Cepeda:** Conceptualization, Methodology, Software, Funding acquisition, Data curation, Writing – original draft. **Sergio García-García:** Data curation, Methodology, Software, Writing – review & editing. **Ignacio Arrese:** . **Francisco Herrero:** Resources, Visualization, Investigation. **Trinidad Escudero:** Resources, Visualization, Investigation. **Tomás Zamora:** Resources, Visualization, Investigation. **Rosario Sarabia:** .

## Data Availability

RHUH-GBM (Original data) (https://www.cancerimagingarchive.net/).
